# Limited knowledge, low risk awareness, and eating out are associated with higher sugar-sweetened beverage consumption among adults aged 18–64 in Beijing

**DOI:** 10.1371/journal.pone.0334416

**Published:** 2025-10-10

**Authors:** Liyu Huang, Yiran Li, Yingjie Yu, Yan Zhang, Bo Yu, Xinyue Guo, Hairong He, Jiali Duan

**Affiliations:** 1 Institute of Nutrition and Food Hygiene, Beijing Center for Disease Prevention and Control, Beijing, China; 2 School of Public Health, Capital Medical University, Beijing, China; 3 School of Public Health, Hebei Medical University, Shijiazhuang, Hebei, China; Yantai Institute of Technology, CHINA

## Abstract

**Objectives:**

The purpose of this study was to analyze the prevalence of sugar-sweetened beverage (SSB) consumption among residents aged 18–64 in Beijing and to identify associated influencing factors.

**Methods:**

A cross-section study was conducted from June 1,2024 to May 31, 2025, including 10,409 residents aged 18–64 in Beijing. Logistic regression was applied to examine demographic, behavioral, and knowledge-related factors associated SSB consumption.

**Results:**

The overall prevalence of SSB consumption was 38.7%. After adjusting for demographic factors, infrequent checking nutrition labels when purchasing food (ORs ranging from 1.348 to 1.570), infrequent active weight monitoring (ORs ranging from 1.290 to 1.428), dining out/taking out food >1 day/week (ORs ranging from3.495 to6.692), moderate-intensity physical activity less than 300 minutes a week (ORs ranging from 1.237 to 1.326), lack of knowledge about SSB (ORs ranging from 1.240 to 1.361), and awareness of the health risks of SSB (OR=1.198) were the risk factors for SSB consumption.

**Conclusions:**

SSB consumption among Beijing adults remains high. Men, urban residents, and younger adults represent priority groups for intervention. Strengthening nutrition education, improving health literacy, and promoting healthier dietary behaviors are essential strategies to reduce SSB consumption and improve population health.

## Introduction

Sugar-sweetened beverages (SSBs) refer to drinks that have any form of sugar added during production and processing, including carbonated drinks, fruit-flavored beverages, tea drinks, energy drinks, and more. The consumption of sugar-sweetened beverages has continued to increase globally and has become an important source of sugar intake in the daily diet of adults. Frequent consumption of SSBs is closely associated with the occurrence of various chronic diseases, such as obesity [1,2], type 2 diabetes [3,4], nonalcoholic fatty liver disease [5,6] and cardiovascular diseases [7], thereby exacerbating the global burden of non-communicable diseases [8,9].

The consumption patterns of sugar-sweetened beverages vary across different countries and regions, with gender, age, and socioeconomic status being significant factors [10]. In 2018, mean global SSB intake was 2.7 servings/week with the largest increase in Sub-Saharan Africa [11]. Total sugar intake declined from 43.6 ± 1.7 mean g/d in 2003–2004 to 22.3 ± 0.8 mean g/d in 2015–2016 among the United States population [12]. The California Health Interview Survey from 2011–2018 found reductions in the annual prevalence and frequency of soda consumption across all age groups and heterogeneous increases in the consumption of fruit drinks among adults and children [13]. Millennials (22–38 years) were twice as likely to drink sweetened fruit juice drinks and energy drinks daily and consumed six times more daily calories from sweetened fruit juice drinks than the other groups in rural Appalachia [14].

In recent years, China has witnessed rising SSB consumption alongside rapid urbanization and lifestyle changes. According to national surveillance data, nearly half of Chinese adults reported consuming sugary beverages, with carbonated drinks being the most common type [15]. Between 1990 and 2019, the number of deaths attributed to SSB intake in China increased by 95%, reaching 46,633 [16]. These figures highlight a growing public health concern. Compared with earlier surveys conducted before 2020, the present study is situated in a post–“Healthy China Action (2019–2030)” era, when national initiatives targeting “three reductions” (salt, oil, and sugar) have been widely implemented [17]. However, despite such measures, up-to-date evidence on adult SSB consumption in metropolitan areas remains limited.

Beijing, as the capital of China and one of the most urbanized megacities, provides a unique context to investigate SSB consumption. It represents both the challenges of rapid socioeconomic transition and the opportunities for public health policy innovation. Previous nationwide studies have described overall SSB intake, but they have rarely focused on large-scale, representative samples in Beijing. Understanding consumption behavior in Beijing is particularly relevant because its population density, dietary patterns, and fast-paced lifestyles may shape beverage choices differently from other Chinese regions. Moreover, Beijing often leads the implementation of health promotion programs, making local data highly valuable for national policymaking.

However, compared to the epidemiological research and intervention practices related to chronic disease risk factors such as oil and salt, there is limited epidemiological research on sugar intake in our country. This study aimed to assess the prevalence of SSB consumption among Beijing adults aged 18–64 and to examine its associations with demographic factors, nutrition-related knowledge, and dietary behaviors. We hypothesized that younger adults, and those with lower health awareness would have higher SSB intake, whereas healthier dietary practices would be protective.

## Materials and methods

### Study population

This study conducted a survey among residents aged 18–64 in Beijing from June 1, 2024 to May 31, 2025. The participants were selected using a multi-stage stratified cluster sampling method: (1) In the 17 districts of Beijing, each district selected 3 streets (the Economic Development Zone selected 2 streets); (2) 2–3 neighborhood committees were selected from each selected street; (3) At least 60 households were surveyed from each neighborhood committee, and only one eligible family member was surveyed from each household. According to equation (1), calculate the required sample size. Based on the results of Chinese Nutrition and Health Knowledge Survey in 2023 awareness rate of residents aged 18–64 in Beijing (43.9%), the minimum sample size of each layer was calculated n = 752 by taking p = 43.9%, δ = 0.1p, μ_α/2_ = 1.96, and deff = 2.5. The sampling is stratified by region (two layers: urban and suburban), age (three layers: 18–34 years, 35–49 years, and 50–64 years), and gender (two layers: male and female). Additionally, taking into account the rate of invalid questionnaires and refusal to participate, the actual sample size is increased by 10%, resulting in a calculated sample size of N = 10,027.


$$n=\frac{{\mu^{\text{2}}}_{\alpha /\text{2}}\times p(\text{1}-p)}{\delta^{\text{2}}}\times \text{deff}$$ (1)

This survey has been approved by the Ethics Committee of the Institute for Nutrition and Health of the Chinese Center for Disease Control and Prevention (Ethics Approval Number: 2022−037), and all participants included in the survey have signed written informed consent.

### Measurements and variable definitions

Chinese Nutrition and Health Knowledge Questionnaire for Adults (CNHKQ) designed uniformly by the Chinese Center for Disease Control and Prevention’s Nutrition and Health Institute was adopted [18]. The content of the questionnaire mainly includes four aspects: basic information, knowledge of nutrition and health, attitudes, and behavioral conditions. This survey was conducted through home visits, where interviewers asked questions face-to-face and filled out the questionnaire based on the respondents’ answers.

The demographic information mainly includes region, gender, age, marital status, education level, occupation, annual income per capita, body mass index (BMI), chronic disease status, etc.

Knowledge related to SSB: (i) Foods or beverages that contain added sugars should be consumed sparingly; (ii) The daily intake of added sugars should not exceed 25 g. Responding ‘yes’ is defined as being aware of this knowledge. If the answer to the question ‘Drinking excessive amounts of SSB may increase the risk of obesity and dental caries’ is yes, it indicates an awareness of the health risks associated with sugary beverages.

Behavioral factors include checking nutrition labels when purchasing food, actively monitoring weight, frequency of dining out/taking out food, moderate-intensity physical activity and frequency of consumption of SSB. Checking nutrition labels when purchasing food: Responses were categorized as “never,” “occasionally,” “sometimes,” “often,” and “always.” Actively monitoring weight: Responses were categorized as “never,” “occasionally,” “sometimes,” “often,” and “always.” Frequency of dining out/taking out food: Responses were categorized as “<1 day/week,” “1-2 days/week,” “3-4 days/week,” “5-6 days/week,” “Every day.” Moderate-intensity physical activity: Responses were categorized as “<150 minutes,” “150~300 minutes,” “≥ 300 minutes.” Frequency of consumption of SSB: Responses were categorized as “<1 day/week,” “1-2 days/week,” “3-4 days/week,” “5-6 days/week,” “Every day.” SSB consumption frequency ≥1 day/week is defined as having SSB consumption behavior.

### Quality control

All investigators underwent standardized training before data collection. Questionnaires were checked on-site by supervisors and re-examined by the municipal quality-control team. For data entry, a double-entry system was applied using EpiData 3.1. Data cleaning followed a structured protocol: (1) Logical checks: Consistency rules were applied (e.g., male participants not reporting pregnancy; implausible combinations such as “never checking nutrition labels” but reporting “always using nutrition labels” were flagged and corrected or excluded). (2) Outliers: Continuous variables such as BMI and age were screened using Z-scores methods. Implausible values were cross-checked against original records; if unresolved, they were set as missing.

### Statistical analyses

All statistical analyses were performed using the statistical software IBM SPSS Statistics version 22.0. The proportion (%) of different demographic groups was described using composition ratios. The inter-group differences were tested using the Chi-square test. Logistic regression analysis was performed to examine factors associated with SSB consumption. The model was adjusted for demographic covariates, including region, gender, age, marital status, education, occupation, annual per capita income, BMI, and presence of chronic disease. Behavioral and knowledge-related variables (checking nutrition labels when purchasing food, actively monitoring weight, dining out/takeout, moderate-intensity physical activity, and awareness variables related to added sugar intake and health risks) were entered simultaneously as independent variables. Odds ratios (ORs) with 95% confidence intervals (CIs) were calculated. Statistical *p*-values were two-sided analyses, with *p* < 0.05 considered statistically significant.

## Results

### Demographic characteristics

A total of 10,409 residents aged 18–64 from Beijing were included in this survey. Among them, 3,273 residents are from urban areas (31.4%), while 7,136 are from suburban areas (68.6%); there are 5,143 male participants (49.4%) and 5,266 female participants (50.6%). The prevalence of overweight and obesity in the surveyed population was relatively high at 51.1%, of which the overweight rate was 36.8% and the obesity rate was 14.3% (Table 1).

**Table 1:** The awareness rate of knowledge related to sugary beverages among residents aged 18 to 64 in Beijing in 2025.

Group	n (%)	Foods or beverages that contain added sugars should be consumed sparingly	*χ* ^*2*^	*P*-Value	The daily intake of added sugars should not exceed 25 g	*χ* ^*2*^	*P*-Value
**Region**			40.772	<0.001		11.056	0.001
Urban	3273 (31.4)	2837 (86.7)			2448 (74.8)		
Suburban	7136 (68.6)	5826 (81.6)			5114 (71.7)		
**Gender**			21.962	<0.001		37.007	<0.001
Male	5143 (49.4)	4191 (81.5)			3598 (70.0)		
Female	5266 (50.6)	4472 (84.9)			3964 (75.3)		
**Age (years)**			13.139	0.011		16.447	0.002
18-24	946 (9.1)	778 (82.2)			640 (67.7)		
25-34	2647 (25.4)	2213 (83.6)			1913 (72.3)		
35-44	2565 (24.6)	2185 (85.2)			1869 (72.9)		
45-54	1773 (17.0)	1465 (82.6)			1325 (74.7)		
55-64	2478 (23.8)	2022 (81.6)			1815 (73.2)		
**Marital status**			1.575	0.455		9.462	0.009
Unmarried	2008 (19.3)	1670 (83.2)			1405 (70.0)		
Married	7912 (76.0)	6596 (83.4)			5792 (73.2)		
Divorced/widowed	489 (4.7)	397 (81.2)			365 (74.6)		
**Education**			75.615	<0.001		17.866	<0.001
Junior high school or below	2323 (22.3)	1816 (78.2)			1609 (69.3)		
High school	2322 (22.3)	1904 (82.0)			1714 (73.8)		
Junior college	2417 (23.2)	2044 (84.6)			1765 (73.0)		
Undergraduate degree or higher	3347 (32.2)	2899 (86.6)			2474 (73.9)		
**Occupation**			10.930	0.012		4.749	0.191
General Occupation	8624 (82.9)	7182 (83.3)			6238 (72.3)		
Healthcare	587 (5.6)	490 (83.5)			446 (76.0)		
Food and Catering	607 (5.8)	481 (79.2)			438 (72.2)		
Education	591 (5.7)	510 (86.3)			440 (74.5)		
**Annual income per capita (RMB: yuan)**			37.911	<0.001		14.482	0.006
< 30000	3277 (31.5)	2627 (80.2)			2307 (70.4)		
30000−40000	2076 (19.9)	1733 (83.5)			1525 (73.5)		
50000-60000	2245 (21.6)	1900 (84.6)			1663 (74.1)		
70000-80000	1002 (9.6)	842 (84.0)			753 (75.1)		
≥ 90000	1809 (17.4)	1561 (86.3)			1314 (72.6)		
**BMI**			0.101	0.992		14.986	0.002
Low body weight	393 (3.8)	326 (83.0)			287 (73.0)		
Normal	4700 (45.2)	3907 (83.1)			3494 (74.3)		
Overweight	3822 (36.8)	3194 (83.4)			2746 (71.7)		
Obesity	1484 (14.3)	1236 (83.3)			1035 (69.7)		
**Suffering from a chronic disease**			20.962	<0.001		4.861	0.088
No	6175 (59.3)	5220 (84.5)			4485 (72.6)		
Yes	2908 (27.9)	2382 (81.9)			2143 (73.7)		
Unclear	1326 (12.7)	1061 (80.0)			934 (70.4)		
**Checking nutrition labels when purchasing food**			84.089	<0.001		168.889	<0.001
Never	699 (6.4)	498 (74.4)			400 (59.8)		
Occasionally	2441 (23.5)	1957 (80.2)			1685 (69.0)		
Sometimes	2623 (25.2)	2175 (82.9)			1818 (69.3)		
Often	3193 (30.7)	2754 (86.3)			2457 (76.9)		
Always	1483 (14.2)	1279 (86.2)			1202 (81.1)		
**Actively monitoring weight**			164.111	<0.001		123.492	<0.001
Never	359 (3.4)	244 (68.0)			210 (58.5)		
Occasionally	2641 (25.4)	2067 (78.3)			1839 (69.6)		
Sometimes	2620 (25.2)	2174 (83.0)			1813 (69.2)		
Often	3679 (35.3)	3226 (87.7)			2805 (76.2)		
Always	1110 (10.7)	952 (85.8)			895 (80.6)		
**Dining out/taking out food**			105.444	<0.001		27.250	<0.001
< 1 day/week	6284 (60.4)	5315 (84.6)			4671 (74.3)		
1–2 days/week	1859 (17.9)	1572 (84.6)			1305 (70.2)		
3–4 days/week	1242 (11.9)	1023 (82.4)			892 (71.8)		
5–6 days/week	692 (6.6)	537 (77.6)			469 (67.8)		
Every day	332 (3.2)	216 (65.1)			469 (67.8)		
**Moderate-intensity physical activity during the week**			2.481	0.289		26.044	<0.001
< 150 minutes	2667 (25.6)	2198 (82.4)			1840 (69.0)		
150 ~ 300 minutes	4277 (41.1)	3557 (83.2)			3188 (74.5)		
≥ 300 minutes	3465 (33.3)	2908 (83.9)			2534 (73.1)		
**Total**	10409 (100.0)	8663 (83.2)			7562 (72.6)		

### The awareness rate of knowledge related to SSB

The awareness rate of the statement that ‘Foods or beverages containing added sugars should be consumed sparingly’ was 83.2%, while the awareness rate for the statement ‘The daily intake of added sugars should not exceed 25 g’ was 72.6%. Overall, participants residing in urban areas, females, with higher education level, higher per capita annual income, frequent reading of nutrition labels when buying food, and lower frequency of eating out or ordering takeout had higher awareness of knowledge of SSB, and the difference was statistically significant (Table 1).

### Awareness of the health risks associated with SSB

84.4% of the participants were aware of the health risks of SSB, believing that drinking too many SSB could increase the risk of obesity and dental caries. Female, 35–44 years old, undergraduate degree or higher, engaged in the education industry, per capita annual income greater than 90,000 yuan, and participants without chronic diseases had a high awareness of the health risks of sugar-sweetened beverages. Individuals who always read nutritional labels when purchasing food, frequently monitor their weight, dine out or order takeout 1–2 days/week, engage in moderate-intensity physical activity for 300 minutes or more per week, and possess knowledge about sugary beverages were more aware of the health risks associated with sugary drinks. The differences were statistically significant (*Ρ* < 0.05). (S1 Table).

### Consumption of SSB

**Fig 1:**
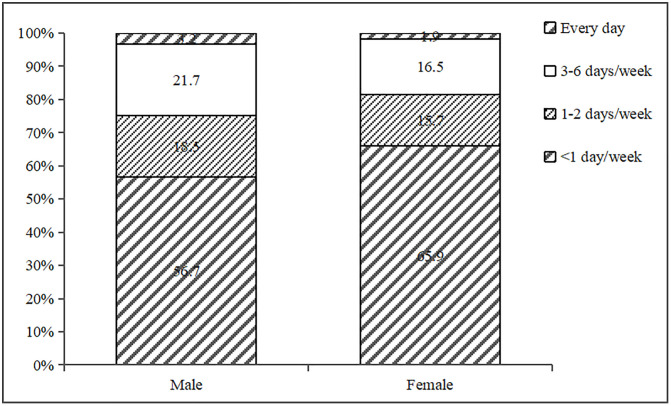
The frequency of SSB consumption among participants of different genders.

**Fig 2:**
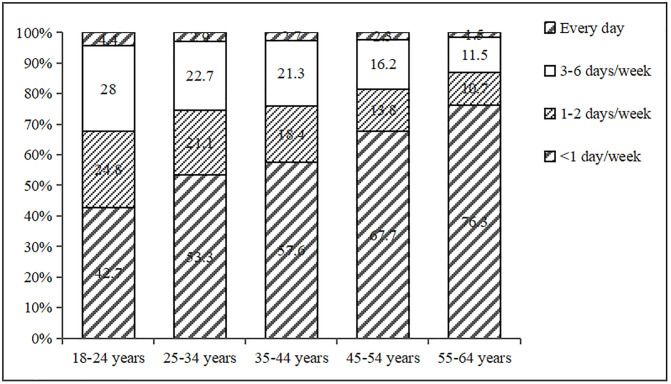
The frequency of SSB consumption among participants of different age groups.

The consumption rate of sugar-sweetened beverages among residents aged 18–64 in Beijing was 38.7%. Male, 18–24 years old, unmarried, higher level of education, higher food and education industry, higher annual income per capita, low body weight, and people who are unclear about chronic diseases are more likely to consume SSB. Participants who always check nutrition labels when purchasing food and actively monitor their weight, dine out or order takeout less than once a week, engage in moderate-intensity physical activity for more than 300 minutes a week, are aware of the knowledge regarding sugary beverages, and possess an awareness of the health risks associated with SSB are less likely to consume sugary beverages (S2 Table). Fig 1 showed the frequency of SSB consumption among participants of different genders, while Fig 2 showed the frequency of SSB consumption among participants of different age groups.

### Multivariate regression analysis of SSB consumption

After adjusting for demographic factors, infrequent checking nutrition labels when purchasing food (OR=1.348, 95%CI: 1.065–1.706; OR=1.570, 95%CI: 1.315–1.875; OR=1.389, 95%CI: 1.171–1.648), infrequent active weight monitoring (OR=1.428, 95%CI: 1.058–1.926; OR=1.334, 95%CI: 1.101–1.615; OR=1.290, 95%CI: 1.069–1.556), dining out/taking out food >1 day/week (OR=3.495, 95%CI: 3.116–3.919; OR=6.307, 95%CI: 5.482–7.257; OR=6.692, 95%CI: 5.585–8.019; OR=6.314, 95%CI: 4.885–8.160), moderate-intensity physical activity less than 300 minutes a week (OR=1.326, 95%CI: 1.178–1.493; OR=1.237, 95%CI: 1.112–1.375), lack of knowledge about SSB (OR=1.361, 95%CI: 1.207–1.535; OR=1.240, 95%CI: 1.122–1.371), and awareness of the health risks of SSB (OR=1.198, 95%CI: 1.058–1.357) were the risk factors for SSB consumption. (Table 2).

**Table 2:** Multivariate logistic regression analysis of SSB consumption behavior among residents aged 18 to 64 in Beijing in 2025.

Group	β	S.E.	Waldχ^2^	OR (95%CI)	*P*值
**Region (Ref: Suburban)**					
Urban	0.204	0.05	16.36	1.226(1.111-1.354)	<0.001
**Gender (Ref: Female)**					
Male	0.253	0.047	28.882	1.287(1.174-1.412)	<0.001
**Age (years) (Ref: 55–64)**					
18-24	0.835	0.118	49.978	2.305(1.829-2.906)	<0.001
25-34	0.52	0.078	44.516	1.682(1.444-1.960)	<0.001
35-44	0.484	0.073	44.426	1.622(1.407-1.870)	<0.001
45-54	0.277	0.077	13.07	1.319(1.135-1.533)	<0.001
**Marital status (Ref: Unmarried)**					
Married	−0.184	0.076	5.819	0.832(0.717-0.966)	0.016
Divorced/widowed	0.017	0.130	0.017	1.017(0.788-1.313)	0.896
**BMI (Ref: Normal)**					
Low body weight	0.207	0.120	2.977	1.230(0.972-1.557)	0.084
Overweight	0.074	0.052	2.008	1.077(0.972-1.193)	0.156
Obesity	0.199	0.071	7.862	1.220(1.062-1.402)	0.005
**Suffering from a chronic disease (Ref: Unclear)**					
No	−0.129	0.070	3.415	0.879(0.767-1.008)	0.065
Yes	−0.223	0.081	7.642	0.800(0.683-0.937)	0.006
**Checking nutrition labels when purchasing food (Ref: Always)**					
Never	0.298	0.120	6.167	1.348(1.065-1.706)	0.013
Occasionally	0.451	0.091	24.816	1.570(1.315-1.875)	<0.001
Sometimes	0.329	0.087	14.260	1.389(1.171-1.648)	<0.001
Often	0.052	0.084	0.381	1.053(0.894-1.240)	0.537
**Actively monitoring weight (Ref: Always)**					
Never	0.356	0.153	5.418	1.428(1.058-1.926)	0.020
Occasionally	0.288	0.098	8.695	1.334(1.101-1.615)	0.003
Sometimes	0.255	0.096	7.072	1.290(1.069-1.556)	0.008
Often	0.014	0.090	0.024	1.014(0.850-1.209)	0.877
**Dining out/taking out food (Ref: < 1 day/week)**					
1–2 days/week	1.251	0.058	457.652	3.495(3.116-3.919)	<0.001
3–4 days/week	1.842	0.072	662.047	6.307(5.482-7.257)	<0.001
5–6 days/week	1.901	0.092	424.413	6.692(5.585-8.019)	<0.001
Every day	1.843	0.131	198.295	6.314(4.885-8.160)	<0.001
**Moderate-intensity physical activity during the week (Ref: ≥ 300 minutes)**					
< 150 minutes	0.282	0.061	21.781	1.326(1.178-1.493)	<0.001
150–300 minutes	0.212	0.054	15.467	1.237(1.112-1.375)	<0.001
**Foods or beverages that contain added sugars should be consumed sparingly (Ref: Yes)**					
No	0.308	0.061	25.228	1.361(1.207-1.535)	<0.001
**The daily intake of added sugars should not exceed 25 g (Ref: Yes)**					
No	0.215	0.051	17.857	1.240(1.122-1.371)	<0.001
**The awareness of health risks associated with SSB (Ref: No)**					
Yes	0.181	0.063	8.121	1.198(1.058-1.357)	0.004

## Discussion

The purpose of this study was to analyze the prevalence of SSB consumption among adult residents aged 18–64 in Beijing and to examine its association with demographic characteristics, nutritional health behaviors, and nutritional health knowledge. The awareness rate of the statement that ‘Foods or beverages containing added sugars should be consumed sparingly’ was 83.2%, while 72.6% of participants were aware that ‘The daily intake of added sugars should not exceed 25 g’. In addition, 84.4% of the participants recognized that excessive SSB intake could increase the risk of obesity and dental caries. Despite this relatively high level of awareness, 38.7% of participants reported SSB consumption ≥1 time per week, and 2.5% consumed SSBs daily. Compared with Western countries, the prevalence reported in our study was lower than that observed in the United States and Australia [19,20]. A global analysis across 187 countries showed that China had relatively low per capita SSB consumption, yet the age- and sex-specific patterns were consistent with worldwide trends, with higher consumption in men and younger adults and declining intake with increasing age [21]. These findings suggest that Beijing is undergoing a nutrition transition, sharing global patterns while still maintaining lower overall prevalence.

Male and younger participants were more likely to consume SSB regularly, a result consistent with findings among Czech adults [11]. Regarding the gender disparity, the higher consumption among males may be influenced by socio-cultural norms. In many societies, including China, sugary drinks are often marketed towards men as a means to replenish energy and are frequently associated with social gatherings and sports events. Our study highlights that young adults, particularly those aged 18–24 years, demonstrated markedly higher SSB intake compared with older adults aged 55–64 years. Similar observations were reported in Saudi Arabia, where younger adults showed a substantially higher risk of SSB consumption compared with older age groups (18–29 years: OR: 4.0, 95% CI [2.6–6.3]; 30–44 years: OR: 2.8, 95% CI [1.8–4.3]) [22]. These cross-national similarities suggest that age-related differences in consumption are not unique to China but reflect broader global behavioral trends [23,24]. Several mechanisms may explain this pattern: young adults may have stronger preferences for sweet taste, greater exposure to SSB marketing, and easier access to affordable beverages in urban environments [25–27]. Moreover, SSBs are often integrated into social activities and dining-out culture among younger populations, further reinforcing consumption. In addition, previous studies indicate that younger individuals may be more susceptible to developing dependency-like behaviors toward SSBs [28,29]. These findings emphasize the importance of designing youth-focused public health interventions to address SSB consumption.

Participants with chronic diseases reported lower SSB consumption compared with those without chronic conditions. Evidence from the National Health Survey in Peru showed that people aware of having diabetes had a lower consumption of both ready-to-drink (0.9 vs. 0.4) and homemade SSB (1.3 vs. 0.8) than those unaware of having diabetes, which is similar to our results [30]. In our study, individuals who were unaware of their chronic illness reported the highest SSB intake. This suggests that disease awareness may promote healthier dietary behaviors through physician counseling, health education, and surveillance conducted in community health centers [31]. Previous studies have also highlighted the effectiveness of tailored educational programs and community-level interventions in promoting healthier eating behaviors among patients with chronic diseases [32,33]. The relationship between SSB consumption and obesity in this population is likely bidirectional. High-calorie, low-satiety SSBs significantly contribute to weight gain, a concern amid Beijing’s nutritional transition. Conversely, obesity may perpetuate high SSB intake through psychological mechanisms like stress-related consumption or entrenched taste preferences. This potential cyclical pattern, where each condition exacerbates the other, underscores a complex public health challenge. Consequently, SSB reduction strategies must be integrated into broader obesity prevention initiatives to effectively break this cycle.

The health knowledge awareness rate of SSB was generally high. The awareness rate of “daily intake of added sugars” in this study was higher than that in Wuhan (21.6%) [34]. This improvement may be attributable to China’s continuous promotion of health promotion actions, such as “three reductions, three health”. Three reductions stand for reducing the intake of salt, oil, and sugar [35,36]. Our results further demonstrated that higher knowledge of SSB-related health risks was protective against consumption. This finding is consistent with prior studies indicating that better diet-related knowledge, attitudes, and behaviors were associated with healthier eating and higher self-rated health among Chinese adults [37,38]. Moreover, knowledge of cardiovascular risks linked to SSB was found to be associated with lower SSB intake [39]. However, our results differ from those of a U.S. study, which reported that although knowledge of health risks varied by demographic characteristics, it was not directly associated with SSB consumption [40]. This discrepancy may reflect a broader phenomenon of a “knowledge–action gap,” whereby individuals are aware of health risks but do not translate this knowledge into behavior change. Evidence from Canada similarly indicates that nutrition knowledge alone may be insufficient to alter dietary habits, as behavioral change is influenced by individual motivation, social support, and environmental factors [41]. These observations suggest that health education should be complemented with supportive environments and policy measures to achieve meaningful reductions in SSB consumption. China could mandate easy-to-understand, front-of-pack warning labels (e.g., “High in Sugar”) as implemented in Chile and Mexico. This empowers consumers to make informed choices at the point of purchase.

Behavioral correlates also play a crucial role. Reading nutrition labels when purchasing food was associated with lower SSB consumption, likely because labels enable individuals to identify healthier options. Previous research has shown that front-of-pack labeling improves consumer understanding of nutritional quality, increases the likelihood of choosing healthier products, and influences purchasing behavior [42–44]. Our study also found that frequent dining out or ordering takeout was significantly associated with higher SSB consumption. This is consistent with findings among Beijing college students, where frequent takeout was associated with higher intake of fat and sugar [45], and with studies in Greece showing that frequent fast-food consumption among children and adolescents was linked to unhealthy dietary patterns characterized by high sugar intake [46]. These findings suggest that the dining-out environment is a critical determinant of SSB intake in urban populations. This phenomenon can be interpreted through several mechanisms. Firstly, SSBs are often the default, heavily promoted, and highly profitable beverage option in restaurants and delivery platforms, creating an environment that nudges consumers towards unhealthy choices. Secondly, meals consumed away from home are often larger in portion size and more energy-dense, with SSBs being a typical component of “value” meals or social dining experiences.

This study has several limitations. First, this study is a cross-sectional investigation, and we are unable to infer causality. Data iterations from future cohort studies may help us define causality. Second, the questionnaire data were self-reported and may be subject to recall bias. We aimed to minimize bias through the use of validated survey tools. Finally, total SSB intake was not captured, and therefore, total intake estimates were lacking in the analysis. In addition, although this study defined the SSB consumption behavior of adults in Beijing, it is limited to generalize the findings to the entire population due to the urban characteristics of the study sample.

## Conclusions

Overall, 38.7% of adults aged 18–64 in Beijing consumed sugary beverages, with a higher prevalence among males than females, and among urban areas compared to suburban areas. Consumption was most common in young adults aged 18–24 years and declined with increasing age. Individuals with chronic illnesses reported lower intake, suggesting greater health awareness. Moreover, healthier nutritional behaviors and higher nutrition-related knowledge were associated with reduced SSB consumption. These findings underscore the need for targeted public health strategies (SSB tax, Construction of Healthy Environments, enhanced labeling) to curb SSB intake. Policy measures should be complemented by health education initiatives that strengthen nutrition literacy and support residents in adopting healthier lifestyles. Our results provide an evidence base to inform tailored interventions for reducing SSB consumption and improving population health.

## Supporting information

S1 Table The awareness of health risks associated with SSB among residents aged 18–64 in Beijing in 2025. (DOCX)

S2 Table Prevalence of SSB consumption among residents aged 18–64 in Beijing in 2025. (DOCX)

S3 Table The frequency of sugar-sweetened beverage consumption among residents aged 18–64 in Beijing in 2025. (DOCX)

S4 Table Multifactor regression analysis of SSB consumption frequency among residents aged 18–64 in Beijing in 2025 (vs < 1 day/week). (DOCX)
