# Supplementary material for: Limited knowledge, low risk awareness, and eating out are associated with higher sugar-sweetened beverage consumption among adults aged 18–64 in Beijing
Source: PLoS One. 2025 Oct 10;20(10):e0334416. doi: 10.1371/journal.pone.0334416 (PMC12513660; doi:10.1371/journal.pone.0334416)
Supplement: S1 Table — (DOCX) [file pone.0334416.s001.docx]

**Supporting information**

**S1 Table. The awareness of health risks associated with SSB among residents aged 18 to 64 in Beijing in 2025.**

| **Group** | **n (%)** | **The awareness of health risks associated with SSB** | | ***χ^2^*** | ***Ρ*-Value** |
| --- | --- | --- | --- | --- | --- |
|  |  | **Yes** | **No** |  |  |
| **Region** |  |  |  | 0.608 | 0.435 |
| Urban | 3273（31.4） | 2777（84.8） | 496（15.2） |  |  |
| Suburban | 7136（68.6） | 6012（84.2） | 1124（15.8） |  |  |
| **Gender** |  |  |  | 52.964 | <0.001 |
| Male | 5143（49.4） | 4208（81.8） | 935（18.2） |  |  |
| Female | 5266（50.6） | 4581（87.0） | 685（13.0） |  |  |
| **Age (years)** |  |  |  | 26.663 | <0.001 |
| 18-24 | 946（9.1） | 804（85.0） | 142（15.0） |  |  |
| 25-34 | 2647（25.4） | 2238（84.5） | 409（15.5） |  |  |
| 35-44 | 2565（24.6） | 2226（86.8） | 339（13.2） |  |  |
| 45-54 | 1773（17.0） | 1500（84.6） | 273（15.4） |  |  |
| 55-64 | 2478（23.8） | 2021（81.6） | 457（18.4） |  |  |
| **Marital status** |  |  |  | 1.938 | 0.379 |
| Unmarried | 2008（19.3） | 1711（85.2） | 297（14.8） |  |  |
| Married | 7912（76.0） | 6673（84.3） | 1239（15.7） |  |  |
| Divorced /widowed | 489（4.7） | 405（82.8） | 84（17.2） |  |  |
| **Education** |  |  |  | 90.909 | <0.001 |
| Junior high school or below | 2323（22.3） | 1847（79.5） | 476（20.5） |  |  |
| High school | 2322（22.3） | 1919（82.6） | 403（17.4） |  |  |
| Junior college | 2417（23.2） | 2063（85.4） | 354（14.6） |  |  |
| Undergraduate degree or higher | 3347（32.2） | 2960（88.4） | 387（11.6） |  |  |
| **Occupation** |  |  |  | 11.116 | 0.011 |
| General Occupation | 8624（82.9） | 7250（84.1） | 1374（15.9） |  |  |
| Healthcare | 587（5.6） | 512（87.2） | 75（12.8） |  |  |
| Food and Catering | 607（5.8） | 506（83.4） | 101（16.6） |  |  |
| Education | 591（5.7） | 521（88.2） | 70（11.8） |  |  |
| **Annual income per capita (RMB: yuan)** |  |  |  | 27.902 | <0.001 |
| <30000 | 3277（31.5） | 2689（82.1） | 588（17.9） |  |  |
| 30000-40000 | 2076（19.9） | 1749（84.2） | 327（15.8） |  |  |
| 50000-60000 | 2245（21.6） | 1913（85.2） | 332（14.8） |  |  |
| 70000-80000 | 1002（9.6） | 858（85.6） | 144（14.4） |  |  |
| ≥90000 | 1809（17.4） | 1580（87.3） | 229（12.9） |  |  |
| **BMI** |  |  |  | 0.880 | 0.830 |
| Low body weight | 393（3.8） | 334（85.0） | 59（15.0） |  |  |
| Normal | 4700（45.2） | 3971（84.5） | 729（15.5） |  |  |
| Overweight | 3822（36.8） | 3222（84.1） | 610（15.9） |  |  |
| Obesity | 1484（14.3） | 1262（85.0） | 222（15.0） |  |  |
| **Suffering from a chronic disease** |  |  |  | 30.897 | <0.001 |
| No | 6175（59.3） | 5314（86.1） | 861（13.9） |  |  |
| Yes | 2908（27.9） | 2395（82.4） | 513（17.6） |  |  |
| Unclear | 1326（12.7） | 1080（81.4） | 246（18.6） |  |  |
| **Checking nutrition labels when purchasing food** |  |  |  | 153.290 | <0.001 |
| Never | 699（6.4） | 515（77.0） | 154（23.0） |  |  |
| Occasionally | 2441（23.5） | 1955（80.1） | 486（19.9） |  |  |
| Sometimes | 2623（25.2） | 2154（82.1） | 469（17.9） |  |  |
| Often | 3193（30.7） | 2825（88.5） | 368（11.5） |  |  |
| Always | 1483（14.2） | 1340（90.4） | 143（9.6） |  |  |
| **Actively monitoring weight** |  |  |  | 226.403 | <0.001 |
| Never | 359（3.4） | 259（72.1） | 100（27.9） |  |  |
| Occasionally | 2641（25.4） | 2070（78.4） | 571（21.6） |  |  |
| Sometimes | 2620（25.2） | 2161（82.5） | 459（17.5） |  |  |
| Often | 3679（35.3） | 3307（89.9） | 372（10.1） |  |  |
| Always | 1110（10.7） | 992（89.4） | 118（10.6） |  |  |
| **Dining out/taking out food** |  |  |  | 50.437 | <0.001 |
| <1 day/week | 6284（60.4） | 5359（85.3） | 925（14.7） |  |  |
| 1-2 days/week | 1859（17.9） | 1616（86.9） | 243（13.1） |  |  |
| 3-4 days/week | 1242（11.9） | 1009（81.2） | 233（18.8） |  |  |
| 5-6 days/week | 692（6.6） | 552（79.8） | 140（20.2） |  |  |
| Every day | 332（3.2） | 253（76.2） | 79（23.8） |  |  |
| **Moderate-intensity physical activity during the week** |  |  |  | 25.030 | <0.001 |
| <150 minutes | 2667（25.6） | 2172（81.4） | 495（18.6） |  |  |
| 150-300 minutes | 4277（41.1） | 3644（85.2） | 633（14.8） |  |  |
| ≥ 300 minutes | 3465（33.3） | 2973（85.8） | 492（14.2） |  |  |
| **Foods or beverages that contain added sugars should be consumed sparingly** |  |  |  | 107.926 | <0.001 |
| No | 1746（16.8） | 1275（73.0） | 471（27.0） |  |  |
| Yes | 8663（83.2） | 7514（86.7） | 1149（13.3） |  |  |
| **The daily intake of added sugars should not exceed 25 g** |  |  |  | 72.017 | <0.001 |
| No | 2847（27.4） | 2264（79.5） | 583（20.5） |  |  |
| Yes | 7562（72.6） | 6525（86.3） | 1037（13.7） |  |  |
| **Total** | 10409（100.0） | 8789（84.4） | 1620（15.6） |  |  |
