# Supplementary material for: Limited knowledge, low risk awareness, and eating out are associated with higher sugar-sweetened beverage consumption among adults aged 18–64 in Beijing
Source: PLoS One. 2025 Oct 10;20(10):e0334416. doi: 10.1371/journal.pone.0334416 (PMC12513660; doi:10.1371/journal.pone.0334416)
Supplement: S2 Table — (DOCX) [file pone.0334416.s002.docx]

**Supporting information**

**S2 Table. Prevalence of SSB consumption among residents aged 18 to 64 in Beijing in 2025.**

| **Group** | **n (%)** | **Prevalence of SSB consumption** | | ***χ^2^*** | ***Ρ*-Value** |
| --- | --- | --- | --- | --- | --- |
|  |  | **Yes** | **No** |  |  |
| **Region** |  |  |  | 0.165 | 0.684 |
| Urban | 3273（31.4） | 1275（39.0） | 1998（61.0） |  |  |
| Suburban | 7136（68.6） | 2750（38.5） | 4386（61.5） |  |  |
| **Gender** |  |  |  | 92.013 | <0.001 |
| Male | 5143（49.4） | 2227（43.3） | 2916（56.7） |  |  |
| Female | 5266（50.6） | 1798（34.1） | 3468（65.9） |  |  |
| **Age (years)** |  |  |  | 488.688 | <0.001 |
| 18-24 | 946（9.1） | 542（57.3） | 404（42.7） |  |  |
| 25-34 | 2647（25.4） | 1235（46.7） | 1412（53.3） |  |  |
| 35-44 | 2565（24.6） | 1088（42.4） | 1477（57.6） |  |  |
| 45-54 | 1773（17.0） | 572（32.3） | 1201（67.7） |  |  |
| 55-64 | 2478（23.8） | 588（23.7） | 1890（76.3） |  |  |
| **Marital status** |  |  |  | 216.677 | <0.001 |
| Unmarried | 2008（19.3） | 1065（53.0） | 943（47.0） |  |  |
| Married | 7912（76.0） | 2785（35.2） | 5172（64.8） |  |  |
| Divorced /widowed | 489（4.7） | 175（35.8） | 314（64.2） |  |  |
| **Education** |  |  |  | 137.604 | <0.001 |
| Junior high school or below | 2323（22.3） | 706（30.4） | 1617（69.6） |  |  |
| High school | 2322（22.3） | 814（35.1） | 1508（64.9） |  |  |
| Junior college | 2417（23.2） | 1025（42.4） | 1392（57.6） |  |  |
| Undergraduate degree or higher | 3347（32.2） | 1480（44.2） | 1867（55.8） |  |  |
| **Occupation** |  |  |  | 17.249 | 0.001 |
| General Occupation | 8624（82.9） | 3263（37.8） | 5361（62.2） |  |  |
| Healthcare | 587（5.6） | 236（40.2） | 351（59.8） |  |  |
| Food and Catering | 607（5.8） | 262（43.2） | 345（56.8） |  |  |
| Education | 591（5.7） | 264（44.7） | 327（55.3） |  |  |
| **Annual income per capita (RMB: yuan)** |  |  |  | 38.325 | <0.001 |
| <30000 | 3277（31.5） | 1163（35.5） | 2114（64.5） |  |  |
| 30000-40000 | 2076（19.9） | 760（36.6） | 1316（63.4） |  |  |
| 50000-60000 | 2245（21.6） | 913（40.7） | 1332（59.3） |  |  |
| 70000-80000 | 1002（9.6） | 410（40.9） | 592（59.1） |  |  |
| ≥90000 | 1809（17.4） | 779（43.1） | 1030（56.9） |  |  |
| **BMI** |  |  |  | 13.966 | 0.003 |
| Low body weight | 393（3.8） | 185（47.1） | 208（52.9） |  |  |
| Normal | 4700（45.2） | 1806（38.4） | 2894（61.6） |  |  |
| Overweight | 3822（36.8） | 1445（37.7） | 2387（62.3） |  |  |
| Obesity | 1484（14.3） | 589（39.7） | 895（60.3） |  |  |
| **Suffering from a chronic disease** |  |  |  | 124.006 | <0.001 |
| No | 6175（59.3） | 2545（41.2） | 3630（58.8） |  |  |
| Yes | 2908（27.9） | 883（30.4） | 2025（69.6） |  |  |
| Unclear | 1326（12.7） | 597（45.0） | 729（55.0） |  |  |
| **Checking nutrition labels when purchasing food** |  |  |  | 301.000 | <0.001 |
| Never | 699（6.4） | 292（43.6） | 377（56.4） |  |  |
| Occasionally | 2441（23.5） | 1188（48.7） | 1253（51.3） |  |  |
| Sometimes | 2623（25.2） | 1151（43.9） | 1472（56.1） |  |  |
| Often | 3193（30.7） | 991（31.0） | 2202（69.0） |  |  |
| Always | 1483（14.2） | 403（27.2） | 1080（72.8） |  |  |
| **Actively monitoring weight** |  |  |  | 264.550 | <0.001 |
| Never | 359（3.4） | 172（47.9） | 187（52.1） |  |  |
| Occasionally | 2641（25.4） | 1249（47.3） | 1392（52.7） |  |  |
| Sometimes | 2620（25.2） | 1127（43.9） | 1471（56.1） |  |  |
| Often | 3679（35.3） | 328（30.6） | 2552（69.4） |  |  |
| Always | 1110（10.7） | 4025（29.5） | 782（70.5） |  |  |
| **Dining out/taking out food** |  |  |  | 1804.228 | <0.001 |
| <1 day/week | 6284（60.4） | 1429（22.7） | 4855（77.3） |  |  |
| 1-2 days/week | 1859（17.9） | 1009（54.3） | 850（45.7） |  |  |
| 3-4 days/week | 1242（11.9） | 859（69.2） | 383（30.8） |  |  |
| 5-6 days/week | 692（6.6） | 490（70.8） | 202（29.2） |  |  |
| Every day | 332（3.2） | 238（71.7） | 94（28.3） |  |  |
| **Moderate-intensity physical activity during the week** |  |  |  | 90.930 | <0.001 |
| <150 minutes | 2667（25.6） | 1198（44.9） | 1469（55.1） |  |  |
| 150-300 minutes | 4277（41.1） | 1682（39.3） | 2595（60.7） |  |  |
| ≥ 300 minutes | 3465（33.3） | 1145（33.0） | 2320（67.0） |  |  |
| **Foods or beverages that contain added sugars should be consumed sparingly** |  |  |  | 76.011 | <0.001 |
| No | 1746（16.8） | 837（47.9） | 909（52.1） |  |  |
| Yes | 8663（83.2） | 3188（36.8） | 5475（63.2） |  |  |
| **The daily intake of added sugars should not exceed 25 g** |  |  |  | 68.354 | <0.001 |
| No | 2847（27.4） | 1284（45.1） | 1563（54.9） |  |  |
| Yes | 7562（72.6） | 2741（36.2） | 4821（63.8） |  |  |
| **The awareness of health risks associated with SSB** |  |  |  | 47.070 | <0.001 |
| No | 1620（15.6） | 750（46.3） | 870（53.7） |  |  |
| Yes | 8789（84.4） | 3275（37.3） | 5514（62.7） |  |  |
| **Total** | 10409（100.0） | 4025（38.7） | 6384（61.3） |  |  |
