# Supplementary material for: Limited knowledge, low risk awareness, and eating out are associated with higher sugar-sweetened beverage consumption among adults aged 18–64 in Beijing
Source: PLoS One. 2025 Oct 10;20(10):e0334416. doi: 10.1371/journal.pone.0334416 (PMC12513660; doi:10.1371/journal.pone.0334416)
Supplement: S3 Table — (DOCX) [file pone.0334416.s003.docx]

**Supporting information**

**S3 Table. The frequency of sugar-sweetened beverage consumption among residents aged 18 to 64 in Beijing in 2025.**

| **Group** | **n (%)** | **<1**  **day/week** | **1-2**  **days/week** | **3-6**  **days/week** | **Every day** | ***χ^2^*** | ***Ρ*-Value** |
| --- | --- | --- | --- | --- | --- | --- | --- |
| **Region** |  |  |  |  |  | 31.980 | <0.001 |
| Urban | 3273（31.4） | 1998（61.0） | 642（19.6） | 547（16.7） | 86（2.6） |  |  |
| Suburban | 7136（68.6） | 4386（61.5） | 1135（15.9） | 1436（20.1） | 179（2.5） |  |  |
| **Gender** |  |  |  |  |  | 99.336 | <0.001 |
| Male | 5143（49.4） | 2916（56.7） | 949（18.5） | 1115（21.7） | 163（3.2） |  |  |
| Female | 5266（50.6） | 3468（65.9） | 828（15.7） | 868（16.5） | 102（1.9） |  |  |
| **Age (years)** |  |  |  |  |  | 492.270 | <0.001 |
| 18-24 | 946（9.1） | 404（42.7） | 235（24.8） | 265（28.0） | 42（4.4） |  |  |
| 25-34 | 2647（25.4） | 1412（53.3） | 558（21.1） | 600（22.7） | 77（2.9） |  |  |
| 35-44 | 2565（24.6） | 1477（57.6） | 473（18.4） | 546（21.3） | 69（2.7） |  |  |
| 45-54 | 1773（17.0） | 1201（67.7） | 245（13.8） | 287（16.2） | 40（2.3） |  |  |
| 55-64 | 2478（23.8） | 1890（76.3） | 266（10.7） | 285（11.5） | 37（1.5） |  |  |
| **Marital status** |  |  |  |  |  | 222.169 | <0.001 |
| Unmarried | 2008（19.3） | 943（47.0） | 492（24.5） | 505（25.1） | 68（3.4） |  |  |
| Married | 7912（76.0） | 5127（64.8） | 1219（15.4） | 1383（17.3） | 183（2.3） |  |  |
| Divorced /widowed | 489（4.7） | 314（64.2） | 66（13.5） | 95（19.4） | 14（2.9） |  |  |
| **Education** |  |  |  |  |  | 161.641 | <0.001 |
| Junior high school or below | 2323（22.3） | 1617（69.6） | 286（12.3） | 375（16.1） | 45（1.9） |  |  |
| High school | 2322（22.3） | 1508（64.9） | 328（14.1） | 422（18.2） | 64（2.8） |  |  |
| Junior college | 2417（23.2） | 1392（57.6） | 441（21.6） | 516（21.3） | 68（2.8） |  |  |
| Undergraduate degree or higher | 3347（32.2） | 1867（55.8） | 722（17.1） | 670（20.0） | 88（2.6） |  |  |
| **Occupation** |  |  |  |  |  | 22.074 | 0.009 |
| General Occupation | 8624（82.9） | 5361（62.2） | 1419（16.5） | 1630（18.9） | 214（2.5） |  |  |
| Healthcare | 587（5.6） | 351（59.8） | 115（19.6） | 107（18.2） | 14（2.4） |  |  |
| Food and Catering | 607（5.8） | 345（56.8） | 117（19.3） | 126（20.8） | 19（3.1） |  |  |
| Education | 591（5.7） | 327（55.3） | 126（21.3） | 120（20.3） | 18（3.0） |  |  |
| **Annual income per capita (RMB: yuan)** |  |  |  |  |  | 65.859 | <0.001 |
| <30000 | 3277（31.5） | 2114（64.5） | 460（14.0） | 606（18.5） | 97（3.0） |  |  |
| 30000-40000 | 2076（19.9） | 1316（63.4） | 332（16.0） | 388（18.7） | 40（1.9） |  |  |
| 50000-60000 | 2245（21.6） | 1332（59.3） | 402（17.9） | 455（20.3） | 56（2.5） |  |  |
| 70000-80000 | 1002（9.6） | 592（59.1） | 198（19.8） | 188（18.8） | 24（2.4） |  |  |
| ≥90000 | 1809（17.4） | 1030（56.9） | 385（21.3） | 346（19.1） | 48（2.7） |  |  |
| **BMI** |  |  |  |  |  | 38.425 | <0.001 |
| Low body weight | 393（3.8） | 2894（61.6） | 830（17.7） | 881（18.7） | 95（2.0） |  |  |
| Normal | 4700（45.2） | 208（52.9） | 82（20.9） | 81（20.6） | 22（5.6） |  |  |
| Overweight | 3822（36.8） | 2387（62.3） | 632（16.5） | 715（18.7） | 98（2.6） |  |  |
| Obesity | 1484（14.3） | 895（60.3） | 233（15.7） | 306（20.6） | 50（3.4） |  |  |
| **Suffering from a chronic disease** |  |  |  |  |  | 170.478 | <0.001 |
| No | 6175（59.3） | 3630（58.8） | 1207（19.5） | 1199（19.4） | 139（2.3） |  |  |
| Yes | 2908（27.9） | 2025（69.6） | 356（12.2） | 462（15.9） | 65（2.2） |  |  |
| Unclear | 1326（12.7） | 729（55.0） | 214（16.1） | 322（24.3） | 61（4.6） |  |  |
| **Checking nutrition labels when purchasing food** |  |  |  |  |  | 368.611 | <0.001 |
| Never | 699（6.4） | 377（56.4） | 98（14.6） | 152（22.7） | 42（6.3） |  |  |
| Occasionally | 2441（23.5） | 1253（51.3） | 489（20.0） | 616（25.2） | 83（3.4） |  |  |
| Sometimes | 2623（25.2） | 1472（56.1） | 560（21.3） | 530（20.2） | 61（2.3） |  |  |
| Often | 3193（30.7） | 2202（69.0） | 469（14.7） | 478（15.0） | 44（1.4） |  |  |
| Always | 1483（14.2） | 1080（72.8） | 161（10.9） | 207（14.0） | 35（2.4） |  |  |
| **Actively monitoring weight** |  |  |  |  |  | 32.956 | <0.001 |
| Never | 359（3.4） | 187（52.1） | 53（14.8） | 89（24.8） | 30（8.4） |  |  |
| Occasionally | 2641（25.4） | 1392（52.7） | 535（20.3） | 626（23.7） | 88（3.3） |  |  |
| Sometimes | 2620（25.2） | 1471（69.4） | 518（19.8） | 569（21.7） | 62（2.4） |  |  |
| Often | 3679（35.3） | 2552（69.4） | 533（14.5） | 535（14.5） | 59（1.6） |  |  |
| Always | 1110（10.7） | 782（70.5） | 138（12.4） | 164（14.8） | 26（2.3） |  |  |
| **Dining out/taking out food** |  |  |  |  |  | 2768.514 | <0.001 |
| <1 day/week | 6284（60.4） | 4855(77.3) | 659(10.5) | 694(11.0) | 76(1.2) |  |  |
| 1-2 days/week | 1859（17.9） | 850(45.7) | 615(33.1) | 369(19.8) | 25(1.3) |  |  |
| 3-6 days/week | 1934(18.6) | 585(30.2) | 453(23.4) | 819(42.3) | 77(4.0) |  |  |
| Every day | 332(3.2) | 94(28.3) | 50(15.1) | 101(30.4) | 87(26.2) |  |  |
| **Moderate-intensity physical activity during the week** |  |  |  |  |  | 111.150 | <0.001 |
| <150 minutes | 2667（25.6） | 1469（55.1） | 474（17.8） | 636（23.8） | 88（3.3） |  |  |
| 150-300 minutes | 4277（41.1） | 2595（60.9） | 749（17.5） | 831（19.4） | 102（2.4） |  |  |
| ≥ 300 minutes | 3465（33.3） | 2320（67.0） | 554（16.0） | 516（14.9） | 75（2.2） |  |  |
| **Foods or beverages that contain added sugars should be consumed sparingly** |  |  |  |  |  | 175.407 | <0.001 |
| No | 1746（16.8） | 909（52.1） | 275（15.8） | 462（26.5） | 100（5.7） |  |  |
| Yes | 8663（83.2） | 5475（63.2） | 1502（17.3） | 1521（17.6） | 165（1.9） |  |  |
| **The daily intake of added sugars should not exceed 25 g** |  |  |  |  |  | 83.961 | <0.001 |
| No | 2847（27.4） | 1563（54.9） | 530（18.6） | 645（22.7） | 109（3.8） |  |  |
| Yes | 7562（72.6） | 4821（63.8） | 1247（16.5） | 1338（17.7） | 156（2.1） |  |  |
| **The awareness of health risks associated with SSB** |  |  |  |  |  | 79.890 | <0.001 |
| No | 1620（15.6） | 870（53.7） | 271（16.7） | 410（25.3） | 69（4.3） |  |  |
| Yes | 8789（84.4） | 5514（62.7） | 1506（17.1） | 1573（17.9） | 196（2.2） |  |  |
| **Total** | 10409（100.0） | 6384（61.3） | 1777（17.1） | 1983（19.1） | 265（2.5） |  |  |
