# Supplementary material for: Limited knowledge, low risk awareness, and eating out are associated with higher sugar-sweetened beverage consumption among adults aged 18–64 in Beijing
Source: PLoS One. 2025 Oct 10;20(10):e0334416. doi: 10.1371/journal.pone.0334416 (PMC12513660; doi:10.1371/journal.pone.0334416)
Supplement: S4 Table — (DOCX) [file pone.0334416.s004.docx]

**Supporting information**

**S4 Table. Multifactor regression analysis of SSB consumption frequency among residents aged 18 to 64 in Beijing in 2025 (vs <1 day/week).**

| **Group** | **1-2 days/week** | | **3-6 days/week** | | **Every day** | |
| --- | --- | --- | --- | --- | --- | --- |
|  | **OR（95%CI）** | ***Ρ*-Value** | **OR（95%CI）** | ***Ρ*-Value** | **OR（95%CI）** | ***Ρ*-Value** |
| **Region (Ref: Urban)** |  |  |  |  |  |  |
| Suburban | 1.036（0.909,1.181） | 0.596 | 1.454（1.270,1.665） | <0.001 | 1.191（0.872,1.627） | 0.271 |
| **Gender (Ref: Male)** |  |  |  |  |  |  |
| Female | 0.807（0.718,0.907） | <0.001 | 0.757（0.675,0.85） | <0.001 | 0.664（0.501,0.879） | 0.004 |
| **Age (years) (Ref: 55-64)** |  |  |  |  |  |  |
| 18-24 | 1.882（1.395,2.540） | <0.001 | 2.810（2.084,3.787） | <0.001 | 3.437（1.733,6.817） | <0.001 |
| 25-34 | 1.494（1.205,1.852） | <0.001 | 1.962（1.586,2.426） | <0.001 | 1.906（1.147,3.168） | 0.013 |
| 35-44 | 1.474（1.209,1.797） | <0.001 | 1.837（1.511,2.233） | <0.001 | 1.740（1.076,2.812） | 0.024 |
| 45-54 | 1.194（0.975,1.461） | 0.086 | 1.442（1.184,1.756） | <0.001 | 1.574（0.964,2.571） | 0.070 |
| **Marital status (Ref: Married)** |  |  |  |  |  |  |
| Unmarried | 1.313（1.096,1.574） | 0.003 | 1.137（0.946,1.368） | 0.172 | 0.835（0.538,1.297） | 0.423 |
| Divorced /widowed | 1.033（0.773,1.380） | 0.826 | 1.360（1.045,1.769） | 0.022 | 1.478（0.811,2.694） | 0.202 |
| **Education (Ref: Undergraduate degree or higher)** |  |  |  |  |  |  |
| Junior high school or below | 0.977（0.797,1.199） | 0.826 | 1.145（0.939,1.398） | 0.181 | 0.898（0.559,1.445） | 0.658 |
| High school | 0.953（0.797,1.138） | 0.593 | 1.164（0.977,1.386） | 0.089 | 1.371（0.923,2.036） | 0.118 |
| Junior college | 0.926（0.795,1.079） | 0.324 | 1.046（0.896,1.221） | 0.570 | 1.141（0.793,1.641） | 0.476 |
| **Occupation (Ref: Education)** |  |  |  |  |  |  |
| General Occupation | 0.914（0.722,1.157） | 0.453 | 1.004（0.786,1.283） | 0.972 | 0.809（0.463,1.412） | 0.455 |
| Healthcare | 0.955（0.695,1.312） | 0.778 | 0.877（0.628,1.225） | 0.441 | 0.696（0.317,1.527） | 0.366 |
| Food and Catering | 1.051（0.760,1.452） | 0.765 | 1.007（0.724,1.402） | 0.966 | 0.819（0.387,1.732） | 0.601 |
| **Annual income per capita (RMB: yuan) (Ref: ≥90000)** |  |  |  |  |  |  |
| <30000 | 0.921（0.766,1.109） | 0.386 | 1.050（0.872,1.266） | 0.605 | 1.144（0.750,1.746） | 0.532 |
| 30000-40000 | 1.014（0.838,1.227） | 0.889 | 1.118（0.920,1.358） | 0.262 | 0.819（0.509,1.318） | 0.410 |
| 50000-60000 | 1.045（0.874,1.250） | 0.630 | 1.168（0.971,1.405） | 0.098 | 1.070（0.693,1.651） | 0.761 |
| 70000-80000 | 0.988（0.797,1.225） | 0.914 | 0.972（0.774,1.219） | 0.804 | 0.892（0.518,1.536） | 0.680 |
| **BMI (Ref: Normal)** |  |  |  |  |  |  |
| Low body weight | 1.172（0.877,1.566） | 0.282 | 1.172（0.871,1.577） | 0.294 | 2.434（1.385,4.278） | 0.002 |
| Overweight | 1.055（0.927,1.200） | 0.414 | 1.073（0.944,1.221） | 0.279 | 1.239（0.903,1.699） | 0.184 |
| Obesity | 1.137（0.950,1.361） | 0.161 | 1.259（1.060,1.496） | 0.009 | 1.646（1.109,2.441） | 0.013 |
| **Suffering from a chronic disease (Ref: Unclear)** |  |  |  |  |  |  |
| No | 1.078（0.903,1.288） | 0.405 | 0.784（0.664,0.924） | 0.004 | 0.556（0.393,0.788） | 0.001 |
| Yes | 0.864（0.700,1.065） | 0.170 | 0.778（0.641,0.944） | 0.011 | 0.610（0.400,0.928） | 0.021 |
| **Checking nutrition labels when purchasing food (Ref: Always)** |  |  |  |  |  |  |
| Never | 1.290（0.939,1.772） | 0.115 | 1.340（1.001,1.793） | 0.049 | 1.429（0.787,2.597） | 0.241 |
| Occasionally | 1.693（1.339,2.140） | <0.001 | 1.533（1.227,1.917） | <0.001 | 1.086（0.650,1.816） | 0.752 |
| Sometimes | 1.746（1.395,2.185） | <0.001 | 1.193（0.960,1.483） | 0.112 | 0.780（0.466,1.307） | 0.346 |
| Often | 1.233（0.991,1.535） | 0.060 | 0.984（0.798,1.214） | 0.880 | 0.527（0.315,0.883） | 0.015 |
| **Actively monitoring weight (Ref: Always)** |  |  |  |  |  |  |
| Never | 1.354（0.905,2.026） | 0.140 | 1.325（0.918,1.912） | 0.1330 | 1.834（0.891,3.775） | 0.099 |
| Occasionally | 1.433（1.117,1.838） | 0.005 | 1.241（0.974,1.581） | 0.081 | 1.302（0.734,2.308） | 0.366 |
| Sometimes | 1.370（1.073,1.748） | 0.011 | 1.226（0.966,1.555） | 0.094 | 1.075（0.604,1.915） | 0.805 |
| Often | 1.084（0.860,1.366） | 0.495 | 0.950（0.758,1.190） | 0.656 | 0.966（0.560,1.668） | 0.902 |
| **Dining out/taking out food (Ref: <1 day/week)** |  |  |  |  |  |  |
| 1-2 days/week | 3.043（2.119,4.368） | <0.001 | 5.835（4.302,7.913） | <0.001 | 38.836（25.980,58.055） | <0.001 |
| 3-6 days/week | 4.525（3.876,5.282） | <0.001 | 8.512（7.384,9.814） | <0.001 | 7.317（5.167,10.361） | <0.001 |
| Every day | 4.406（3.834,5.063） | <0.001 | 2.742（2.351,3.199） | <0.001 | 1.691（1.056,2.707） | 0.029 |
| **Moderate-intensity physical activity during the week (Ref: ≥300 minutes)** |  |  |  |  |  |  |
| <150 minutes | 1.128（0.970,1.311） | 0.117 | 1.557（1.342,1.806） | <0.001 | 1.329（0.940,1.879） | 0.108 |
| 150-300 minutes | 1.146（1.004,1.309） | 0.043 | 1.358（1.186,1.555） | <0.001 | 1.147（0.826,1.593） | 0.413 |
| **Foods or beverages that contain added sugars should be consumed sparingly (Ref: Yes)** |  |  |  |  |  |  |
| No | 1.061（0.905,1.244） | 0.464 | 1.264（1.117,1.429） | <0.001 | 1.713（1.299,2.259） | <0.001 |
| **The daily intake of added sugars should not exceed 25 g (Ref: Yes)** |  |  |  |  |  |  |
| No | 1.166（1.028,1.322） | 0.017 | 1.544（1.338,1.782） | <0.001 | 2.352（1.753,3.155） | <0.001 |
| **The awareness of health risks associated with SSB (Ref: No)** |  |  |  |  |  |  |
| Yes | 1.060（0.902,1.246） | 0.479 | 1.293（1.114,1.501） | 0.001 | 1.410（1.022,1.946） | 0.037 |
